# The current status of job burnout among online nurses delivering Internet+ home care services and influencing factors: a cross-sectional study

**DOI:** 10.3389/fpubh.2026.1779629

**Published:** 2026-03-06

**Authors:** Yinan Wang, Fangfang Jin, Wenhong Su, Ruru Guo, Jingjing Wang

**Affiliations:** 1 Community Nursing Teaching and Research Section, School of Nursing & Health, Zhengzhou University, Zhengzhou, China; 2 Department of Radiotherapy and Chemotherapy for Oncology, First Affiliated Hospital of Ningbo University, Ningbo, China; 3 Ward 2, Department of Hematology, The First Affiliated Hospital of Ningbo University, Ningbo, China

**Keywords:** coping style, home visiting nurse, Internet + nursing service, job burnout, pressure

## Abstract

**Background:**

Nurse burnout is prevalent. Most existing studies focus on specialized units—such as ICUs and emergency departments. In China, “Internet+” home care is primarily delivered part-time by clinical nurses from tertiary hospitals. Yet, stress coping strategies and burnout among these nurses remain underexplored. This study examines the relationship between coping styles and burnout in this context.

**Method:**

A cross-sectional design was used. From September to November 2024, a total of 311 nurses from seven tertiary hospitals who performed “internet +” services were surveyed, of whom 287 completed valid questionnaires and were included in the final analysis. The relationship between stress coping styles and job burnout levels was analyzed using the Simplified Coping Style Questionnaire (SCSQ) and the Nurse Burnout Scale (NBS).

**Result:**

Nurses reported low job burnout scores (122.29 ± 28.46; ranging 57–228), indicating either extremely mild or no job burnout. Nurses who employed positive or negative coping strategies reported scores of 22.03 ± 7.56 (range 0–36) and 9.56 ± 5.01 (range 0–24), respectively. Specifically, positive coping styles were negatively correlated with total job burnout scores (*r* = −0.415, *p* < 0.01), whereas negative coping styles were positively correlated with total scores (*r* = 0.174, *p* < 0.01). Factors that influence job burnout include nurses' clinical department, educational background, marital status, average monthly income from “internet +” nursing services over the past three months, and coping styles. Married nurses with higher academic qualifications working in outpatient and emergency care departments experienced notably lower levels of job burnout.

**Conclusion:**

The stress coping styles of nurses who provide “internet +” nursing services in tertiary hospitals and job burnout levels are significantly correlated. Nursing managers should focus on stress coping strategies and job burnout awareness to create environments in which positive coping strategies are encouraged. This approach may reduce job burnout among nurses in tertiary hospitals while promoting their overall physical and mental wellbeing. Enhancing nurses' job satisfaction and happiness with “internet +” nursing services can improve work quality, reduce turnover, and meet the increasing demand for nursing and health services.

## 1 Introduction

With the aging of the global population, there is a growing demand for home-based care and support services for older adults (1). However, survey data from the World Health Organization (WHO) indicate that by 2024, the shortage of nurses reached approximately 4.6 million (2). This significant deficit presents challenges to healthcare systems worldwide (3). In China, since 2019, the National Health Commission has implemented an “internet +” nursing service model that allows patients to order home care services online via a mobile app (4). Advancements in internet-based medical care have allowed clinical nurses to perform part-time “internet +” nursing services outside of traditional workplaces. Home nursing services have become essential supplements within nursing and healthcare services (5, 6). In Western countries, full-time nurses typically provide home care; in contrast, nurses employed at tertiary general hospitals in China often perform comprehensive Internet + nursing services only part-time (7, 8). With this approach, nurses have additional work responsibilities, which may increase work-related pressures and psychological burdens.

Job burnout is a psychological syndrome resulting from prolonged emotional and interpersonal stressors in the workplace. It represents an extreme reaction when individuals struggle to manage work-related pressures, manifesting as emotional, attitudinal, and behavioral exhaustion due to sustained stress exposure (9). The prevalence of job burnout among nurses globally is alarmingly high. Woo et al. (10) conducted a survey among 45,539 nurses across 49 countries in 2020 and revealed an incidence rate of 11.23%. Job burnout adversely affects the physical and mental wellbeing of nursing staff and is correlated with care quality and patient safety, significantly contributing to nurse turnover (11, 12). This issue is particularly pronounced among nurses that provide “Internet +” nursing services within tertiary hospitals. Compared with traditional hospital-based nursing roles, “Internet +” nursing services are typically delivered in non-clinical settings and often involve additional responsibilities beyond routine duties, including travel and care provision in unfamiliar environments. These work characteristics may intensify emotional and physical demands and thereby increase susceptibility to burnout. Nurses providing home care face numerous stressors not only from their primary workplace responsibilities but also from unique challenges related to their part-time roles. Research has shown that these nurses often encounter risks such as workplace violence (verbal abuse, physical altercations, and sexual harassment), feelings of isolation at work, unfamiliar environments, and traffic accidents (13–15). These additional pressures exacerbate job burnout levels. Moreover, excessive workloads lead to physical discomfort, such as muscle strain, and other issues that further worsen job burnout while also negatively impacting overall health (16). In tertiary hospitals, nurses are required to provide part-time internet-based nursing services alongside their primary duties, hindering their ability to cope effectively with these demands.

Coping styles, also known as coping mechanisms, refer to the cognitive and behavioral strategies individuals use when facing setbacks and pressure, as conceptualized in the transactional model of stress and coping proposed by Lazarus and Folkman. These coping styles play crucial roles in mediating and moderating psychological stress (17). An individual's coping style influences both the nature and intensity of their stress response, thereby affecting the relationship between stress and its outcomes. Positive coping styles can effectively limit the negative impact of stress on mental health, whereas negative coping styles may increase stress perception. Within the transactional stress-coping framework, coping styles are regarded as key mediators between perceived stress and job burnout (18, 19). Research has indicated that prolonged use of emotion-oriented coping strategies can lead to emotional exhaustion (20), which is a core component of job burnout. Online nurses face various multidimensional stressors, such as high workload, role ambiguity, and technical adaptation challenges (14). In particular, Shimizutani et al. (21) noted that job burnout among nurses stems from not only their work environment but also their personal traits and coping styles. When confronted with pressure, nurses do not merely accept external influences; instead, they adapt through cognitive and emotional regulation processes. They may employ positive strategies such as seeking support or applying problem-solving plans or resort to negative strategies such as avoidance or denial. In tertiary hospitals in which nurses provide “Internet +” nursing services, nurses exhibit different coping methods owing to various pressures (22). Positive coping styles are associated with increased adaptability and stress management capability and reduced job burnout; conversely, negative coping strategies can exacerbate these issues (18, 19).

In China, the development of “Internet +” nursing services' has led researchers to focus primarily on nurses' willingness to provide these services and the safety and quality of patient care. However, the coping styles of nursing staff who provide “internet +” nursing services under work pressure and the extent of nurses' job burnout have been overlooked.

This article investigates part-time “internet +” nursing service providers in tertiary hospitals in Ningbo, Zhejiang Province, to explore the relationship between their job burnout levels and coping styles in relation to work pressure. The goal of this study is to provide a theoretical basis for understanding the mental health status of these nurses and optimizing human resource management among nurses in tertiary hospitals.

## 2 Methods

### 2.1 Study design

A cross-sectional design was employed in this study, with data collected from September to November 2024. An online questionnaire was created, and the automatically generated QR codes were shared with nursing administrative departments at seven tertiary hospitals in Ningbo city, thereby facilitating anonymous participation by nurses. The electronic questionnaire began with a statement outlining the study's purpose, assuring respondents of the anonymity of their responses and their ability to withdraw at any time. In addition, the respondents were informed that reading the instructions and answering the questions constituted informed consent. Contact information for the principal investigator was included for respondents seeking further information about the research. Nurses were required to complete all the questions before submitting their responses.

### 2.2 Participants

Ningbo city is one of the first six pilot regions in China in which “Internet +” nursing services have been implemented, with an annual volume of nearly 100,000 instances. Most participating nurses are clinical staff from tertiary grade A hospitals, reflecting the region's specific model for these services (4, 22).

***Inclusion criteria*:** (a) Professional qualifications with ≥5 years of experience or a nursing title; (b) a clinical nurse at a tertiary hospital in Ningbo city; (c) provided home care services within the past month; and (d) able to read and understand the questionnaire content.

***Exclusion criteria*:** (a) Nurses not on duty (due to study or vacation); (b) poor professional conduct or complaints against the nurse; and (c) participation in a similar study within the past year. This study was approved by the Life Science Ethics Review Committee of Zhengzhou University.

### 2.3 Research tools

***Social Demographic Characteristics Questionnaire:*** This questionnaire consists of 14 items, including the nurses' sex, age, years of service, department, marital status, education level, professional title, employment type, monthly income, income from “Internet +” nursing services, order acceptance frequency per month, service duration (including travel time), distance traveled for home nursing services, and whether they are specialized nurses.

***The Simplified Coping Style Questionnaire (SCSQ):*** The SCSQ, which was validated by Xie (23), is based on the “Coping Style Questionnaire” developed by Folkman (24) and has been widely used in China. It consists of two subscales aimed at evaluating positive coping strategies (items 1–12, 12 items) and negative coping strategies (items 13–20, 8 items). Responses are scored as follows: 0 = no adoption, 1 = occasional adoption, 2 = sometimes adopted, and 3 = frequently adopted. The scores for each subscale are calculated separately; higher scores indicate more frequent use of that coping strategy. Positive coping and negative coping were scored separately according to the two SCSQ subscales. To describe the dominant coping style at the individual level, Z scores were calculated for the positive and negative subscale scores (Z_positive and Z_negative). A coping tendency index was then computed as Z_positive-Z_negative. Participants were classified as positive-dominant when the index was >0 and negative-dominant when the index was ≤ 0. The Cronbach's α co-efficients were as follows: the overall internal consistency was 0.90, the positive response subscale score was 0.89, and the negative response subscale score was 0.78.

***The Nurse Burnout Scale (NBS):*** The NBS, which was revised by Song et al. (25), was specifically designed to assess job burnout among nurses. It consists of five dimensions, namely, environmental stressors, burnout, positive personality traits, coping strategies, and burnout outcomes, totaling 57 items. A Likert scale from 1 to 4 was used: 1 indicated complete disagreement, 2 indicated basic disagreement, 3 indicated basic agreement, and 4 indicated complete agreement. Items 25–27, 31, 32, 35, 36, 39, and 41–43 were reverse scored. Higher total scores indicate more severe burnout levels: a score ≤ 140.5 indicates no or very mild burnout; a score of 140.6 to 160.4 indicates mild to moderate burnout; and a score ≥160.5 indicates severe burnout. The overall Cronbach's α coefficient of the scale is 0.92; the subscale coefficients range from 0.638 to 0.863.

### 2.4 Quality control

According to Kendall's sample size estimation method (26), the questionnaire included 21 analytical variables. To determine the required sample size, we multiplied the number of variables by 10 and accounted for a 20% invalid response rate, resulting in a total sample size of 252 individuals. Data collection was conducted using electronic questionnaires with “allow breakpoint sequential answering” enabled. Only fully completed questionnaires could be submitted; respondents would jump directly to unanswered questions until all were addressed. Each account ID was permitted only one submission. After the original data were exported as Excel files, any responses with clearly contradictory answers were removed.

### 2.5 Statistical methods

The original data from the online questionnaire were exported as Excel files and analyzed using IBM SPSS 25.0 software. Normality was assessed through skewness and kurtosis; Normality was assessed by converting skewness and kurtosis to Z values (Z = statistic/SE). An absolute Z value > 1.96 (*p* < 0.05) or > 2.58 (*p* < 0.01) indicates a significant deviation from normality (27). The sociodemographic characteristics of the nurses providing “Internet +” nursing services in tertiary hospitals are presented as numbers (N) and percentages (%). Continuous variables are expressed as the mean (M) ± standard deviation (SD). Pearson bivariate correlation analysis was used to examine the relationship between nurses' stress coping styles and job burnout, whereas multiple linear regression analysis was used to identify factors influencing job burnout among nurses. A *p* value < 0.05 was considered to indicate statistical significance.

## 3 Results

### 3.1 Sociodemographic characteristics of the participants

A total of 311 nurses participated in the survey, yielding 287 valid responses, for an effective recovery rate of 92.28%. Among them, 284 (98.95%) of the respondents were female. In addition, 97 (33.80%) and 117 (40.77%) nurses were in internal medicine and surgery departments, respectively. Most participants were married 259 (90.24%). In terms of education level, 275 (95.82%) of the respondents had a bachelor's degree, whereas 6 (2.09%) had a master's degree or higher. In terms of income from online booking services over the past three months, 242 nurses (84.32%) reported earning less than 400 yuan monthly; most nurses received fewer than four orders per month (226; 78.75%). The average service duration was ≤ 120 min for 238 respondents (82.93%), and the average distance traveled to provide services was less than 12 kilometers for 217 individuals (75.61%). Additionally, 73 (25.44%) and 214 nurses (74.56%) were classified as specialized and non-specialized, respectively (Table 1).

**Table 1:** Sociodemographic data of nurses providing “Internet +” nursing services in tertiary hospitals [*N* (%)].

**Variable**		***N***	**%**
Sex	Male	3	1.05
	Female	284	98.95
Age (years)	21–30	31	10.80
	31–40	176	61.32
	41–50	78	27.18
	51–60	2	0.70
Work experience (years)	5–10 years	60	20.91
	11–20 years	178	62.02
	>20 years	49	17.07
Department	Internal medicine	97	33.80
	Surgery	117	40.77
	Other	73	25.43
Marital status	Unmarried	26	9.06
	Married	259	90.24
	Divorced	2	0.70
Educational background	Secondary vocational school	1	0.35
	Junior college	5	1.74
	Bachelor's degree	275	95.82
	Master's degree or above	6	2.09
Professional title	Nurse	76	26.48
	Senior nurse	176	61.32
	Associate chief nurse	32	11.15
	Chief nurse	3	1.05
Employment type	Public institution (established position)	251	87.46
	Contractual/non–established Position	36	12.54
Monthly income (RMB)	<5,000	16	5.57
	5,000–9,999	215	74.91
	≥10,000	56	19.52
Average monthly income from online nursing services (past 3 months, RMB)	<400	242	84.32
	400–800	34	11.85
	>800	11	3.83
Average monthly order volume of online nursing services (past 3 months)	<4	226	78.75
	4	24	8.34
	5–7	20	7.00
	≥8	17	5.91
Average monthly duration of online nursing services (including travel time, minutes)	≤120	238	82.93
	121–210	30	10.45
	>210	19	6.62
Average monthly travel distance for online nursing services (kilometers)	≤12	217	75.61
	13–20	49	17.07
	>20	21	7.32
Is the nurse a certified specialist nurse?	Yes	73	25.44
	No	214	74.56

Values are presented for descriptive purposes only. Categories with small sample sizes were not included in between-group comparisons.

### 3.2 Descriptive statistics on the stress coping styles and job burnout levels of nurses providing “Internet +” nursing services in tertiary hospitals

The SCSQ questionnaire was used to investigate the stress coping styles of nurses who provide “internet +” nursing services in tertiary hospitals. The results showed that 133 nurses (46.30%) adopted positive coping styles and 154 (53.70%) adopted negative coping styles. The score for the positive coping style was 22.03 ± 7.56 (range 0–36), and the score for the negative coping style was 9.56 ± 5.01 (range 0–24). Coping style type (positive-dominant vs. negative-dominant) was classified based on the comparison of standardized scores of the two subscales. The coping tendency index (Z_positive-Z_negative) was 0.00 ± 1.10 (range −3.00 to 2.96); a value > 0 indicates positive-dominant coping, whereas a value ≤ 0 indicates negative-dominant coping. The total job burnout score for nurses who provided “internet +” nursing services in tertiary hospitals was 122.29 ± 28.46 (range 57–228), which indicated a relatively low level of job burnout (Table 2).

**Table 2:** Stress coping styles and job burnout levels of nurses providing “Internet +” nursing services in tertiary hospitals (*N* = 287).

**Variables**	**Possible range**	**Minimum**	**Maximum**	**Mean**	**SD**
Positive coping score	0–36	0.00	36.00	22.03	7.56
Negative coping score	0–24	0.00	24.00	9.56	5.01
Coping tendency index (Z_positive–Z_negative)	−3 to 3	−3.00	2.96	0.00	1.10
Environmental stressor score	15–60	15.00	59.00	32.10	8.37
Burnout score	9–36	9.00	35.00	18.39	6.47
Positive personality trait score	11–44	11.00	40.00	23.77	5.31
Coping strategy score	8–32	8.00	23.00	16.84	2.86
Burnout consequence score	14–64	14.00	56.00	31.18	10.52
Total nurse burnout score	57–228	59.00	199.00	122.29	28.46

SD, standard deviation.

### 3.3 Correlation analysis of stress coping styles and job burnout among nurses providing “Internet +” nursing services in tertiary hospitals

Pearson correlation analysis was conducted to examine the relationship between stress coping styles and job burnout among nurses providing “Internet +” nursing services in tertiary hospitals. The results indicated that online nurses employed positive coping styles when facing stress (*r* = −0.415, *p* < 0.01) and exhibited various types of coping strategies (*r* = −0.537, *p* < 0.01). In contrast, negative coping styles were positively correlated with job burnout (*r* = 0.174, *p* < 0.01) (Table 3).

**Table 3:** Correlation analysis of stress coping styles and job burnout levels of nurses providing “Internet +” nursing services in tertiary hospitals.

**Variables**	**Positive coping style**	**Negative coping style**	**Coping style type**	**Occupational burnout among nurses**
Positive coping style	1			
Negative coping style	0.398^**^	1		
Coping style type	0.549^**^	−0.549^**^	1	
Occupational burnout among nurses	−0.415^**^	0.174^**^	−0.537^**^	1

Coping style type was derived from the coping tendency index (Z_positive-Z_negative): >0 = positive-dominant, ≤ 0 = negative-dominant. ^**^p < 0.01 (2 tail).

### 3.4 Analysis of factors influencing job burnout among nurses providing “Internet +” nursing services in tertiary hospitals

The factors that influence job burnout among nurses were examined using job burnout as the dependent variable and sociodemographic data and coping styles as independent variables for multiple linear regression analysis. The key findings indicate that clinical department, educational background, marital status, average monthly income from online nursing services provided over the past three months, and coping styles significantly influence job burnout (*p* < 0.05), explaining 36.7% of the variance (R2 = 0.367, *F* = 16.007, *p* = 0.000). Nurses in outpatient and emergency departments reported lower job burnout scores than those in internal medicine and surgery departments did (B = −9.259, *p* = 0.012). Additionally, higher educational levels were correlated with lower burnout scores (B = −27.637, *p* = 0.005), (B = −30.147, *p* = 0.025). Compared with unmarried nurses, married nurses experienced less burnout (B = −9.607, *p* = 0.048). For each point increase in positive coping, job burnout decreased by 2.006 points (B = −2.006, *p* < 0.001). Conversely, for each point increase in negative coping, job burnout increased by 2.059 points (B = 2.059, *p* < 0.001) (Table 4).

**Table 4:** Multivariate analysis of factors influencing job burnout for nurses providing “Internet +” nursing services in tertiary hospitals.

**Variables**	**Unstandardized co-efficient**		**Standardized co-efficient**	***t***	***p***	***R^2^***	***F***
	**B**	**SE**	**β**				
(Constant)	194.501	12.695		15.321	0.000	0.367	16.007 *p* = 0.000
**Department (Ref: internal medicine)**							
Surgery	−2.231	3.244	−0.039	−0.688	0.492		
Other	−9.259	3.648	−0.142	−2.538	**0.012**		
**Marital status (Ref: unmarried)**							
Married	−9.607	4.830	−0.100	−1.989	**0.048**		
Divorced	−4.077	17.313	−0.012	−0.235	0.814		
**Educational level (Ref: junior college or below)**							
Bachelor's degree	−27.637	9.691	−0.195	−2.852	**0.005**		
Master's degree or above	−30.147	13.393	−0.152	−2.251	**0.025**		
**Monthly online nursing income (Ref: past 3 months**<**400 RMB)**							
400–800 RMB	−4.132	4.338	−0.047	−0.953	0.342		
>800 RMB	−14.167	7.391	−0.096	−1.917	**0.050**		
Positive coping style	−2.006	0.201	−0.533	−9.960	**0.000**		
Negative coping style	2.059	0.306	0.362	6.730	**0.000**		

Dependent variable: job burnout for nurses providing “internet +” nursing services in tertiary hospitals. The bold values indicates “*P* = 0.05”

## 4 Discussion

This cross-sectional study found a significant association between stress coping strategies and job burnout among nurses who provide “Internet +” nursing services in tertiary hospitals (*r* = −0.537, *p* < 0.01). A positive coping style was also negatively correlated with job burnout (*r* = −0.415, *p* < 0.01), while a negative coping style showed a positive correlation (*r* = 0.174, *p* < 0.01). The survey results indicated that job burnout among nurses who were engaged in Internet + nursing services in Ningbo City, Zhejiang Province, was relatively low. Factors that influenced nurses' job burnout included their clinical department, educational background, marital status, average monthly income from online nursing services over the past three months, and coping style. These factors explained 36.7% of the variation (*R*^2^ = 0.367, *F* = 16.007, *p* = 0.000).

The survey showed that nearly half of the nurses adopted constructive coping styles, although the overall level of positive coping remained relatively low. At the same time, more than half of the nurses predominantly relied on negative coping strategies. From the perspective of stress-coping theory, this pattern may be associated with differences in individual resources, personality traits, and behavioral tendencies. Previous studies have demonstrated that individuals with greater personal and environmental resources are more likely to use positive coping strategies and less likely to adopt negative ones (28). Similar findings have been reported among ICU nurses, where open-minded and extroverted individuals tended to employ positive coping strategies (29), and among Polish nurses, where better physical and mental health was associated with greater use of task-oriented coping under stress (30). The ambiguous overall coping tendency observed in this study further reflects the heterogeneity of coping responses among nurses providing “Internet +” nursing services when facing work-related pressure. Currently, standardized guidelines for coping strategies in this context are lacking, highlighting the need for future research to develop targeted and scenario-specific coping interventions.

The overall job burnout level among nurses providing “Internet +” nursing services in tertiary hospitals was relatively low. This finding may be partly associated with a moderate workload, as most nurses reported a limited number of service orders over the past three months. Previous studies have shown that excessive workloads increase burnout and reduce service quality, whereas appropriate workloads are associated with greater workplace satisfaction (31, 32). In addition, organizational support provided through the service platform, such as flexible task allocation and logistical assistance may help reduce practical burdens and improve work-life balance (33). These supportive measures may contribute to mitigating burnout and promoting both physical and psychological wellbeing among nurses engaged in “Internet +” nursing services.

The level of job burnout among nurses providing “Internet +” nursing services in tertiary hospitals was correlated with their stress coping styles (*r* = −0.537, *p* < 0.01). In particular, job burnout was negatively correlated with the use of positive coping styles (*r* = −0.415, *p* < 0.01) and positively correlated with the use of negative coping styles (*r* = 0.174, *p* < 0.01), which is consistent with Kupcewicz and Józwik's (34) research. A problem-centered positive coping approach involves reassessing actions under stress, making timely adjustments, and adapting strategies through methods such as seeking social support, which is associated with lower perceived stress and greater support resources, and has been linked to lower levels of job burnout and higher job satisfaction in previous studies. Conversely, emotion-focused negative coping strategies that involve avoidance or excessive reliance on others can lead to increased job burnout.

Several demographic and work-related factors were associated with job burnout among nurses providing “Internet +” nursing services, including clinical department, educational background, marital status, average monthly income from these services, and coping styles (*p* < 0.05). Nurses working in outpatient and emergency departments reported lower burnout levels, which differs from the findings of Xie et al. (35). This discrepancy may be related to differences in workload characteristics and coping patterns, as emergency nurses often adopt more positive coping strategies under high-intensity conditions (36). In contrast, nurses in internal medicine and surgery departments experience sustained physical demands and complex daily tasks, which may reduce their willingness to engage in additional “Internet +” services.

Nurses with higher educational attainment tended to report lower burnout levels, consistent with previous studies (37), possibly due to stronger professional identity, greater self-efficacy, and enhanced adaptability derived from systematic training and experience (38). Married nurses also exhibited lower burnout levels, which may be associated with greater work experience and stronger family support (39, 40). In addition, higher income from “Internet +” nursing services was associated with lower burnout, potentially reflecting the motivating role of financial incentives, particularly for nurses facing economic pressures (41).

In this study, we examined the mental health status of nurses providing “Internet +” nursing services in tertiary hospitals in Ningbo City, representing one of the first investigations focusing specifically on job burnout in this emerging service context. The sample size was relatively large and provided a reasonable representation of nurses engaged in “Internet +” nursing services. However, several limitations should be acknowledged. First, this study was based on a single-center, cross-sectional design; therefore, causal relationships between coping styles and job burnout cannot be inferred. Second, all data were collected using self-report questionnaires, which may be subject to self-report bias, including recall bias and social desirability bias. Third, the study was conducted within a specific cultural and institutional context in China, and the findings may not be directly generalizable to nurses working in different healthcare systems or cultural settings. Future studies employing multi-center designs, larger samples, longitudinal follow-up, and qualitative approaches are warranted to further elucidate the dynamics of job burnout and stress coping strategies among nurses engaged in “Internet +” nursing services.

## 5 Conclusions

This study investigates job burnout levels and stress coping styles among online nurses in tertiary hospitals and analyzes the factors that influence job burnout. The survey results indicate that job burnout among these nurses is relatively mild. They employ various coping methods to manage the work-related pressures of online nursing services. The analysis identifies sociodemographic and job characteristics as influencing factors of job burnout, including the clinical department, educational background, marital status, average monthly income from online nursing over the past three months, and coping styles. This information serves as a reference for nursing managers and policymakers. The “internet +” nursing service will contribute significantly to public health in China, and nursing staff are essential to this effort. Ensuring their physical and mental wellbeing is vital for stabilizing the nursing team and promoting the sustainable development of “internet + nursing” services.

## Data Availability

The original contributions presented in the study are included in the article/supplementary material, further inquiries can be directed to the corresponding author.
